# Supplementary material for: The Haptoglobin Response after Aneurysmal Subarachnoid Haemorrhage
Source: Int J Mol Sci. 2023 Nov 29;24(23):16922. doi: 10.3390/ijms242316922 (PMC10707007; doi:10.3390/ijms242316922)
Supplement: Supplementary file 1 [file ijms-24-16922-s001.zip › ijms-2720414-supplementary.pdf]

Supplementary Table 1 – Association between cerebrospinal fluid cytokines and serum haptoglobin levels by haptoglobin phenotype

Cytokines (pg/mL)	Hp 1-1 (95% CI)	P value	Hp 2-1 (95% CI)	P value	Hp 2-2 (95% CI)	P value
IFN-gamma	11.591 (-1.965 – 25.148)	0.088	1.862 (-2.012 – 5.736)	0.336	-4.396 (-15.360 – 6.569)	0.415
IL-1beta	1.413 (-0.136 – 2.961)	0.071	**0.815 (0.300 – 1.329)**	**0.003**	-0.312 (-1.405 – 0.782)	0.561
IL-10	1.271 (-0.131 – 2.672)	0.073	**0.548 (0.155 – 0.941)**	**0.008**	-3.956 (-10.815 – 2.903)	0.245
IL-12p70	**9.796 (0.266 – 19.326)**	**0.045**	**2.588 (0.717 – 4.460)**	**0.008**	0.278 (-6.448 – 7.005)	0.933
IL-13	9.833 (-6.088 – 25.753)	0.208	**9.352 (4.326 – 14.378)**	**0.001**	-8.209 (-17.853 – 1.436)	0.092
IL-2	1.214 (-0.373 – 2.801)	0.124	**0.560 (0.163 – 0.957)**	**0.007**	-0.585 (-1.556 – 0.386)	0.225
IL-4	**8.570 (0.013 – 17.128)**	**0.050**	**2.328 (1.049 – 3.606)**	**0.001**	-0.105 (-5.793 – 5.584)	0.970
IL-6	**3057.935 (71.648 – 6044.221)**	**0.045**	**666.453 (102.085 – 1230.820)**	**0.022**	69.399 (-1917.844 – 2056.643)	0.943
IL-8	408.761 (-407 – 1224.612)	0.302	**492.841 (17.816 – 967.866)**	**0.042**	-82.987 (-852.504 – 686.530)	0.825
TNF-alpha	2.023 (-1.123 – 5.170)	0.191	**1.516 (0.329 – 2.703)**	**0.014**	**-2.707 (-5.261 – -0.153)**	**0.039**
Principal Component 1	**1.850 (0.119 – 3.581)**	**0.038**	**0.809 (0.353 – 1.264)**	**0.001**	-0.578 (-1.631 – 0.475)	0.268
Principal Component 2	-0.482 (-1.534 – 0.570)	0.344	0.220 (-0.060 – 0.501)	0.120	-0.412 (-1.218 – 0.393)	0.301
Principal Component 3	-0.078 (-0.249 – 0.094)	0.350	-0.038 (-0.101 – 0.025)	0.227	-0.248 (-1.099 – 0.604)	0.553
